# Cost-effectiveness of health-related lifestyle advice delivered by peer or lay advisors: synthesis of evidence from a systematic review

**DOI:** 10.1186/1478-7547-11-30

**Published:** 2013-12-04

**Authors:** Mark Pennington, Shelina Visram, Cam Donaldson, Martin White, Monique Lhussier, Katherine Deane, Natalie Forster, Susan M Carr

**Affiliations:** 1 Health Economics, London School of Hygiene & Tropical Medicine, 15-17 Tavistock Place, London WC1H 9SH, UK; 2 Centre for Public Policy and Health (CPPH), School of Medicine, Pharmacy and Health, Wolfson Research Institute for Health and Wellbeing, Durham University Queen’s Campus, Stockton-on-Tees TS17 6BH, UK; 3 Yunus Centre for Social Business & Health, Glasgow Caledonian University, Level 3 - Buchanan House, 58 Port Dundas Road, Glasgow G4 0BA, UK; 4 Institute of Health & Society/Fuse UKCRC Centre for Translational Research in Public Health, Newcastle University, Baddiley-Clark Building, Faculty of Medical Sciences, Newcastle University, Newcastle upon Tyne NE2 4AX, UK; 5 Faculty of Health & Life Sciences/Fuse UKCRC Centre for Translational Research in Public Health, Room H012, Coach Lane Campus East, Northumbria University, Newcastle-upon-Tyne NE7 7XA, UK; 6 School of Nursing Sciences, Faculty of Medicine and Health Sciences, Edith Cavell Building, University of East Anglia, Norwich NR4 7TJ, UK

**Keywords:** Health-related lifestyle advisor, Peer counsellor, Cost-effectiveness

## Abstract

**Background:**

Development of new peer or lay health-related lifestyle advisor (HRLA) roles is one response to the need to enhance public engagement in, and improve cost-effectiveness of, health improvement interventions. This article synthesises evidence on the cost-effectiveness of HRLA interventions aimed at adults in developed countries, derived from the first systematic review of the effectiveness, cost-effectiveness, equity and acceptability of different types of HRLA role.

**Methods:**

The best available evidence on the cost-effectiveness of HRLA interventions was obtained using systematic searches of 20 electronic databases and key journals, as well as searches of the grey literature and the internet. Interventions were classified according to the primary health behaviour targeted and intervention costs were estimated where necessary. Lifetime health gains were estimated (in quality-adjusted life years, where possible), based on evidence of effectiveness of HRLAs in combination with published estimates of the lifetime health gains resulting from lifestyle changes, and assumptions over relapse. Incremental cost-effectiveness ratios are reported.

**Results:**

Evidence of the cost-effectiveness of HRLAs was identified from 24 trials included in the systematic review. The interventions were grouped into eight areas. We found little evidence of effectiveness of HRLAs for promotion of exercise/improved diets. Where HRLAs were effective cost-effectiveness varied considerably: Incremental Cost effectiveness Ratios were estimated at £6,000 for smoking cessation; £14,000 for a telephone based type 2 diabetes management; and £250,000 or greater for promotion of mammography attendance and for HIV prevention amongst drug users. We lacked sufficient evidence to estimate ICERs for breastfeeding promotion and mental health promotion, or to assess the impact of HRLAs on health inequalities.

**Conclusions:**

Overall, there is limited evidence suggesting that HRLAs are cost-effective in terms of changing health-related knowledge, behaviours or health outcomes. The evidence that does exist indicates that HRLAs are only cost-effective when they target behaviours likely to have a large impact on overall health-related quality of life. Further development of HRLA interventions needs to target specific population health needs where potential exists for significant improvement, and include rigorous evaluation to ensure that HRLAs provide sufficient value for money.

## Introduction

Behaviour is recognised as a key determinant of health, with modifiable lifestyle factors such as smoking, physical inactivity, poor diet and alcohol consumption, contributing significantly to morbidity and mortality [1]. This is particularly the case in developed countries, where these major health risks tend to be more prevalent amongst the lower socio-economic groups and therefore contribute to social inequalities in health [2,3]. Evidence from the UK indicates that the decline in the proportion of the population that engages in unhealthy behaviours has been mainly in higher socio-economic groups resulting in widening inequalities [4]. The UK government has responded with public health policy setting out the action needed to encourage and enable individuals to make healthier lifestyle choices, with a particular focus on those living in socio-economically disadvantaged areas [5].

Awareness of the role of behavioural factors in health has increased in the general population in developed countries, along with a growing chronic disease burden [6]. Healthcare systems have responded with a shift from paternalistic to partnership models of care, and sought to promote increased involvement of patients and the wider public [7]. The expansion of roles to deliver health-related lifestyle advice represents one strategy in this new partnership approach. An example from the UK is the National Health Service (NHS) health trainer role, which aims to empower people to adopt healthy behaviours by offering practical support from someone who understands the pressures they face [5]. Similar types of lay or peer-based health-related lifestyle advisor (HRLA) role have been widely used in many contexts [8-11].

Previous reviews have suggested that HRLAs may be effective in improving access to health services and addressing health inequalities, in part by providing cultural leverage [12,13]. Whilst evidence on cost-effectiveness is lacking, a perception exists that interventions involving trained but unqualified peer or lay advisers are cheaper than those involving health professionals. However, there are often hidden costs associated with coordination, training and supervision for these roles [14]. We therefore set out to examine systematically the evidence on the effectiveness of HRLAs and to determine in which areas they are likely to be cost-effective. Specifically, we were interested in HLRAs working in the developed world, where contexts differ significantly from those facing HRLAs in developing countries and where modifiable lifestyle behaviours pose a greater threat to health, as noted above. The results reported here are part of a larger evidence synthesis commissioned by the National Institute for Health Research (NIHR) Health Technology Assessment (HTA) Programme, which considered both qualitative and quantitative evidence for the effectiveness, equity and acceptability of different types of HRLA [15].

## Methods

### Literature search

Full details of the search for Medline are provided in Additional file 1 and details of strategies for the other databases are available from the full report [15]. The literature searches included a combination of HRLA, health improvement and methodological terms. The PICOS (Population, Interventions, Comparators, Outcomes and Study Designs) framework was used to generate search terms. Studies were included if they described interventions that were: delivered in developed countries by trained but generally unqualified HRLAs (paid workers or volunteers); consisted of education, training, support or counselling aimed at individuals or groups of peers with the explicit aim of health improvement; and were iterative (rather than a one-off event) and delivered in person, by telephone, post, online or electronically. Studies were excluded if they described (rather than evaluated) an intervention, related to acute care or a single episode of advice-giving, did not have the explicit aim of health improvement, or were not published in English. We also excluded studies focusing solely on advice or training delivered to children or adolescents, as there is already an established literature on peer education for young people. We included all studies with quantitative data on relevant interventions rather than restrict the review to full economic evaluations.

Articles published up to and including September 2008 were searched in the following bibliographic databases: ArticleFirst, ASSIA, British Humanities Index, Cinahl, DARE, Embase, Francis, IBSS, Medline, NHS Economic Evaluation Database, PAIS, Psycinfo, Science Citation Index, Social Science Citation Index, Social Services Abstracts, Sociological Abstracts, SIRS researcher, Web of Knowledge, Worldcat, Zetoc. The search string was modified in accordance with the functions available on each database. Grey literaturewas retrieved through searches using the Google search engine, with the first 100 results retrieved by each search strategy scanned for relevance; searches of specific websites; and contacting experts working in the field to request suggestions for relevant literature. Citation searches of the Science Citation Index and the Social Science Citation Index were carried out in order to identify all citations of those studies assessed as relevant. The reference lists of papers identified as relevant to the review were also checked.

Studies were assessed by two reviewers for their methodological rigour based on the Quality Assessment Tool for Quantitative Studies [16] and included in the review when they were rated as being of strong or moderate quality overall. Any disagreement between the reviewers was resolved through discussion to achieve consensus or, where required, review by a third member of the research team. Full details of the literature search are available in the published report [15].

### Data analysis and synthesis

The disparate nature of the included studies precluded a meta-analysis of study findings. Instead, evidence on the effectiveness of HRLAs was assessed based on: i) the quality and nature of the studies; and ii) the target population. Where evidence existed on the cost-effectiveness of HRLA interventions we evaluated it. Generally this evidence was lacking and so we synthesised our own estimate of cost-effectiveness. We first grouped interventions according to the behaviour targeted as this dictated the size of the potential health gain. Within groups we then considered whether the reported interventions used similar or different modes of delivery. Typical modes of delivery included: community activities and mailshots (considered low intensity); telephone and group counselling (low to medium intensity); and personalised counselling (high intensity). Where studies in the same target behaviour group used the same mode of delivery, we selected one study to inform estimates of costs and effectiveness based on rigor of study design and relevance of the study population. Where interventions used a different mode of delivery, we compared them as alternative strategies, either against no intervention, or against non-HRLA delivered alternatives where these would be readily available. We ranked interventions according to effectiveness and eliminated dominated and extendedly dominated options prior to calculating incremental cost-effectiveness ratios (ICERs) [17]. The perspective of included studies was rarely stated, but none documented costs arising outside of the health sector. Where we synthesized cost-effectiveness estimates we considered only health benefits and costs falling on the health sector.

Our method built on previous work which examined the cost-effectiveness of interventions to incentivise physician activities which improve patients’ health [18]. This study recognised that the total cost of such activities include the cost of the intervention and the impact on overall health costs of the change in patient health. Hence the traditional formula for calculating the ICER can be rewritten to express changes in costs as the sum of these two components. Few studies report long term health gains. However, estimates of the long term health gain from a change in underlying behaviour may be available. If we assume long term health gains are proportional to the magnitude or extent of behaviour change the health gain attributable to the intervention can be expressed as a product of the change in outcomes measured in patients and the health gain attributable to a unit change in outcome. The ICER can then be expressed as:

$$\begin{array}{ll} \mathrm{ICER} & =\Delta C/\Delta Q=\left(\Delta C_{i}+\Delta C_{\mathrm{bh}}\right)/\Delta Q\\ & =\left(\Delta C_{i}+\Delta C_{\mathrm{bh}}\right)/\Delta Q_{\mathrm{bh}}{.e}_{i} \end{array}$$

where ΔC is the change in overall (health) costs, ΔQ is the change in outcome attributable to the intervention, ΔC_i_ is the cost of the intervention, ΔC_bh_ is the overall change in health care costs from the underlying behaviour change, ΔQ_bh_ is the health gain attributable to a unit change in behaviour, and e_i_ is the mean proportion or degree of change achieved by the intervention population.

We determined e_i_ from the primary outcome reported in each study. These were typically changes in behaviour (such as mammography attendance) or in physiological markers (such as HbA1c concentration). We assessed assumptions underpinning analysis in each study and substituted conservative results where assumptions may have been generous (e.g. we assumed smoking cessation participants lost to follow up had resumed smoking). We searched the published literature for modelling studies reporting the lifetime health gains in Quality Adjusted Life Years [19] (QALYs) (ΔQ_bh_) and health care cost impacts (ΔC_bh_) attributable to changes in behaviour or associated with changes in physiological health markers. Where more than one modelling study was available we selected the study based on quality and applicability to a UK context.

Intervention costs (ΔC_i_) were taken from cost data reported in the studies reviewed, where these were available. Costs in foreign currency were converted to UK pounds and inflated to 2008 prices. In the absence of cost data we estimated programme costs. We estimated the total staff time to deliver an intervention, and applied a unit cost which included training and overheads from an authoritative UK source (Unit Costs of Health and Social Care) for an occupation that most closely matched the activity described in the intervention [20]. Details of the costs estimations are given in Additional file 1.

Little evidence is available on long-term recidivism for lifestyle change interventions [21,22]. The exception to this is smoking cessation, where recidivism is substantial [23]. Seven year follow-up of participants in a lifestyle intervention for diabetes management showed that the majority had relapsed [24]. We assumed that interventions would be repeated in the third, sixth and tenth year after the initial intervention, and that despite this 50% of participants achieving change at follow-up would subsequently relapse gaining no health benefits. Costs for implementing the intervention in years three, six and ten were added to the implementation costs in the first year after discounting at 3.5% per annum to estimate the total cost of the intervention. We did not assume that smoking cessation interventions would be repeated; instead, we assumed that 75% of abstainers after three months would subsequently relapse based on data from 1990 California Tobacco Survey [25]. We did not assume that HIV prevention work amongst drug users would be repeated in subsequent years, given the transient nature of the population; instead, we applied conventional, conservative assumptions that intervention effects lasted for the duration of observation only.

Where estimated ICERs were likely to be sensitive to assumptions around health gains or recidivism, we tested these assumptions in sensitivity analysis.

## Results

**Figure 1:**
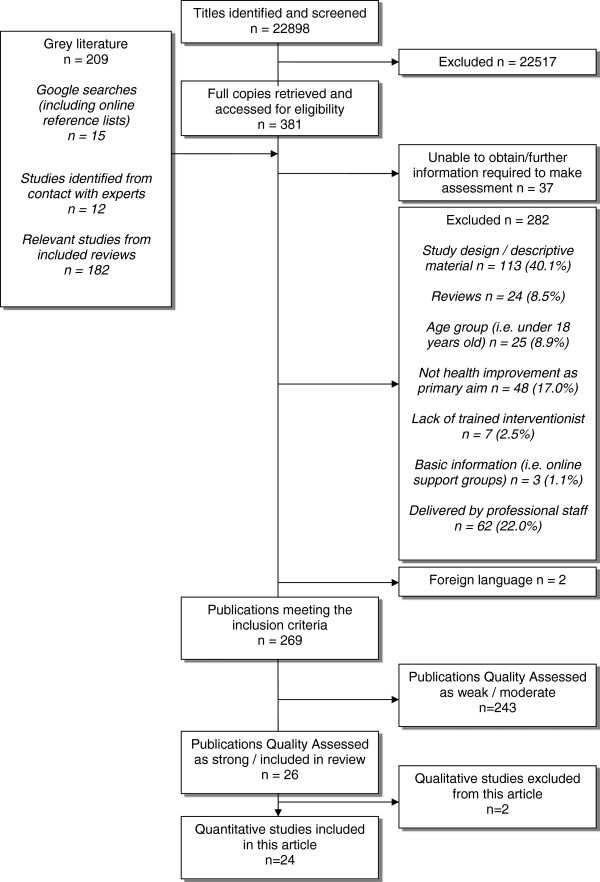
Flowchart showing study selection process.

After quality appraisal 24 met the inclusion criteria for the review. Figure 1 illustrates the search results and screening process.

Interventions included in the review were grouped into eight health areas: general chronic disease management; management of type 2 diabetes; promotion of smoking cessation; breast cancer screening; prevention of HIV infection; healthy diet and/or physical activity; breastfeeding; and mental health. Each of these areas is described in detail below.

### General chronic disease management

Five studies were reviewed on the subject of lay-led disease management for a range of chronic conditions [26-30]. Three of these [26-28] were conducted in the UK, including an evaluation of the national Expert Patients Programme [26]. All of the studies reported significant improvements in patient self-efficacy and self care behaviour attributable to the intervention. Barlow *et al.*[27] demonstrated statistically significant improvements in anxiety and depression. Kennedy *et al.*[26] reported improvements in physical health, Lorig *et al.* (2003) [29] found significant improvements in pain, and both studies reported improvements in psychological wellbeing and health distress. There was some evidence that health improvements were maintained over time. The extent to which improvements in self-efficacy and symptom management translate into gains in health-related quality of life (HRQoL) is unclear, although the impact of chronic diseases on HRQoL is likely to be considerable.

Evidence of the impact of interventions on health care utilisation was inconsistent. Kennedy *et al.*[26] reported reductions in both primary and secondary care attributable to the intervention. The differences were not statistically significant, but a reduction in inpatient days in the intervention arm was sufficient to offset the intervention cost. Griffiths *et al.*[28] and Barlow *et al.*[27] found no impact on health care utilisation attributable to the intervention. Lorig *et al.* (1999) [30] found a reduction in inpatient days but no reduction in primary care. Lorig *et al.* (2003) [29] found a reduction in primary care and emergency admissions, but no change in inpatient days.

#### Estimating cost-effectiveness

Richardson *et al*. undertook a cost-effectiveness analysis of the Expert Patients Programme, and reported a 94% probability that it was cost-effective at a threshold of £20,000 per QALY [31]. This analysis appears to be robust, but may lack generalizability. Most of the intervention costs (£250 per patient) were offset by reductions in inpatient days, attributable to a few resource-intensive patients. Evidence from the other studies of the impact on secondary care is mixed. Nevertheless, the chronic disease management programmes offer the possibility of enabling patient empowerment at modest or zero overall cost.

### Management of type 2 diabetes

Three studies reported different methods of delivering lay-led lifestyle and disease management advice to poor, urban patients with diabetes [32-34]. Young *et al.*[32] describes a low intensity telephone counselling intervention, Gary *et al.*[33] reports a group counselling intervention delivered by promotoras (a term for lay health advisers used by Spanish-speaking communities) and Lujan *et al.*[34] reports an individual counselling intervention. We analysed each intervention as an alternative approach to promoting diabetes disease management in marginalised populations. Change in HbA1c concentration, a well-recognised physiological marker of diabetes control [35], was the primary outcome of each intervention. All three studies reported falls in HbA1c levels of similar magnitude attributable to the intervention: 0.25% (95% CI 0.08 to 0.73) in Lujan *et al.*; 0.31% (95% CI 0.11 to 0.52) in Young *et al.*; and 0.30% (CI −0.18 to 0.78) in Gary *et al.* The cost of the telephone-based intervention in Young *et al.* (£258 per participant) is reported separately [36]. We estimated costs per participant of £757 and £988 for Gary *et al.* and Lujan *et al.* respectively. On the basis of these costs the telephone counselling intervention dominates the other two (it is cheaper and more effective). However, differences in baseline HbA1c between intervention and control in Lujan *et al.* may have diminished the observed treatment effect through regression to the mean. Consequently, we applied a favourable assumption that the treatment effect in Lujan *et al.* is the fall in HbA1c in the treatment arm between baseline and follow-up (0.45%).

#### Estimating cost-effectiveness

We assumed a linear relationship between changes in HbA1c concentration and the resulting impact on health gains and costs. A number of studies have estimated the health gain in QALYs from improved control of HbA1c [37-42]. We applied a value of 0.38 QALYs per 1% fall in HbA1c concentration taken from the Center for Outcomes Research (CORE) diabetes model [37]. Estimates of the costs saved from a fall in HbA1c levels in a UK setting were taken from the Diabforecaster model (£686 2008 GBP for a fall from 8.0% to 7.0%) [38]. In the base case analysis, the ICER for the telephone counselling intervention in Young *et al.* was £13,600. The community health worker intervention in Gary *et al.* is dominated by the telephone counselling intervention. The ICER for the promotora intervention in Lujan *et al.* is £94,500.

In a sensitivity analysis the number of patients who relapse (and gain no health improvement) was varied between 25% and 75%. This is mathematically equivalent to assuming a QALY gain from HbA1c control of plus or minus 50% (0.19 and 0.57 QALYs). The resulting ICER for Young *et al.* varied from £8,400 to £28,800, and the ICER for Gary *et al.* varied from £62,400 to £191,000. Hence, the conclusion that a low intensity intervention for the management of type 2 diabetes is cost-effective at thresholds acceptable in the UKis robust to variation in health gains or recidivism.

An additional study estimated an ICER at £43,400 for the intervention described in Young *et al.* and concluded that the intervention was not cost-effective [36]. The analysis used data from the Centers for Disease Control type 2 diabetes model, in which US style intensive management of HbA1c levels generate considerable additional health care costs from a fall in HbA1c [39]. Under UK practice style, the estimated overall health care costs attributable to a fall in HbA1c levels are negative, which would greatly reduce the ICER for the intervention in Young 2005.

### Smoking cessation

Four reviewed studies focused on advice or counselling regarding smoking cessation [43-46]. The evidence in May *et al.*[43] suggests that a ‘buddy system’ based on mutual support is not effective. The population in Emmons *et al.* were cancer survivors which limits the generalizability of the study [44]. The population in Woodruff *et al.*[45] appears representative of individual, ethnically-targeted smoking cessation services. Consequently, we considered this intervention, and assumed a three month quit rate of 17.4% based on the intention-to-treat analysis rather than the primary results (which ignored those lost to follow-up). We estimated the cost of the intervention at £215 per participant.

Estimates of the average QALY gain for cessation from smoking by Fiscella and Franks [47], and Cromwell [48] concur (1.98 and 1.97 QALYs respectively), although recent evidence [49] would suggest both are conservative. Health care costs related to smoking are considerable [50]. The impact of smoking cessation on health care costs unrelated to smoking is less well established, but these will inevitably rise with increased longevity and may outweigh the direct cost savings [51]. We applied a conservative assumption that the overall cost savings were zero. We assumed that 50% of three-month abstainers would have relapsed at one year and that 50% of one-year abstainers would subsequently relapse, with all those who relapse gaining no health benefits. Hence 25% of three-month quitters were assumed to have quit permanently, gaining 1.97 QALYs each.

#### Estimating cost-effectiveness

We compared the HRLA intervention, to reasonable alternatives available. We assumed that 3% of smokers who wish to stop are successful without seeking professional support [52]. The success rate of brief advice in conjunction with Nicotine Replacement Therapy or Buproprion was estimated at 4%, [53] with an estimated cost of £47 [54]. Pharmacy-based smoking cessation services were assumed to deliver a 5% quit rate at £55 per participant [55]. We assumed an annual success rate of 10% for smokers’ clinics and costs per participant of £350 [55].

Cost-effectiveness calculations are presented in Table 1. The data indicate that smokers’ clinics are more effective than individual counselling from HRLAs, although costs are similar. Smokers’ clinics are cost-effective and the preferred option. However, this intensive group-based therapy may not appeal to many smokers. Tailoring services to smokers’ choices is likely to boost motivation and resulting quit rates. Hence, HRLA interventions might be cost-effective as an alternative service. Compared with willpower alone, the ICER for smoking cessation advice delivered by HRLAs is £3800.

**Table 1:** Cost-effectiveness calculations for various smoking cessation options

	**Cost (£)**	**Annual quit rate**	**QALY gain**	**Incremental gain (QALYs)**	**Incremental cost (£)**	**ICER**
Willpower alone	0	3%	0.0295	-	-	-
Brief advice	47	4%	0.0396	0.0099	47	Extendedly dominated
Pharmacy services	55	5%	0.0495	0.0099	8	£2,800
Tailored HRLA counselling	215	8.7%	0.0857	0.0364	160	£4,400
Smokers’ clinics	350	10%	0.0985	0.0128	135	£10,500

### Mammography promotion

Four studies evaluated HRLA interventions to promote breast cancer screening (mammography) [56-59]. Three studies [56-58] targeted broadly similar populations of poor, rural women, whereas the fourth [59] targeted recent immigrants to the US. The likelihood of specific barriers to mammography in non-native language speaking communities limits the generalizability of the latter study. Hence we considered two types of intervention: a low intensity intervention focusing on community events and mass mailshots as described in Andersen *et al.*[56], and a high intensity intervention based primarily on individual counselling as described by Earp *et al.*[57] and Paskett *et al*. [58] The treatment effect (2.5% uptake) and costs ($34 per participant, £34 2008 GBP) reported for the community arm in Andersen *et al.* were assumed for the low intensity intervention, and the treatment effect (15.2% uptake) and costs ($730 per participant, £657 2008 GBP) reported in Paskett *et al.* for the high intensity intervention.

A number of authors have modelled the cost-effectiveness of mammography to determine the optimum screening frequency/age range [60-65]. Recent modelling studies have reported lifetime benefits of mammography in the range 0.0324–0.0386 QALYs for biennial or triennial screening [60-62]. We used the estimate of 0.0386 QALYs from Rojnik *et al.*[60] which is generous, and matches UK screening policy (triennial from 50 to 70 years of age). Overall health care costs from mammography of €191 (£148 2008 GBP) were taken from the same study.

#### Estimating cost-effectiveness

The ICER for the low-intensity intervention was £251,000 and the ICER for the higher intensity intervention was £896,000. Sensitivity analysis assuming 0% relapse without repeating the HRLA intervention generated an ICER for the low intensity intervention of £39,000. Andersen *et al.*[56] reports a cost-effectiveness analysis of the community activity intervention, giving a cost per life-year saved of US$56,000 (1995 US$) or £56,000 (2008 GBP). Description of the analysis is scant and no mention is made of any allowance for recidivism. Nevertheless, the analysis supports the conclusion that the HRLA intervention would not be considered cost-effective in the UK.

### HIV prevention

Two reviewed studies describe the training of illegal drug users to deliver HIV prevention messages, primarily promoting condom use and the sterilisation of drug injection equipment [66,67]. Whilst both studies are mainly qualitative, quantitative data on each intervention has also been published [68,69]. The two interventions are similar, but Dickson-Gomez *et al.* (2003) [66] included a control group who received an appropriate comparison intervention, and therefore analysis was based on that study. The study reports reduction in both risky sexual and injection behaviours. However, an ordinal scale was used to define the magnitude of risky behaviours obfuscating the absolute reduction following the intervention, and the authors elected to ignore any reported increases in risky behaviour which may have biased results.

#### Estimating cost-effectiveness

Data in Latkin *et al.* indicates the intervention was more effective in reducing needle-sharing than unsafe sex [68]. We considered only the impact of reductions in needle-sharing given that the risk of infection from shared needles is higher than from unprotected vaginal sex (although not unprotected anal sex) [70]. We based estimates of infections avoided on a Bernoulli-process model of transmission, where probability of infection is a function of the number of unsafe acts, the risk of transmission from an unsafe act, and the general prevalence of disease (equation 1 below) [71]. We applied the standard assumptions that the observed reduction in risk behaviour occurs only for the duration of the intervention, and that persons prevented from infection are not subsequently infected.

(1) $$\begin{array}{l} \mathrm{Probability}\;\mathrm{of}\;\mathrm{infection}\;P\\ \;=\;1\;‒\;{\left\{\left(1‒\pi \right)\;+\;\pi \left(1\;-\;\left(a_{d}\cdot a_{i}\right)\right)\right\}}^{n} \end{array}$$

where π = HIV prevalence

α_d_ = risk of infection of needle used by sero-positive user = 0.9 [72].

α_i_ = risk of infection from infected needle = 0.0067 [73].

n = number of incidences of needle sharing

We considered a London setting where increased HIV prevalence of 4% [74] amongst drug users would render the programme more effective than in the rest of England (prevalence 0.6% [75]). We estimated 0.00722 cases of HIV infection avoided for the intervention and 0.00288 for the control, an incremental gain from the intervention of 0.004332 cases averted (details in Additional file 1).

We assumed that averting one case of HIV infection avoided £143,000 [76] and generated a health gain of around 5.37 QALYs [77]. On that basis, the intervention saves £619 and generates 0.0233 QALYs compared to the control, or £1,032 and 0.0388 QALYs compared to no intervention. Total intervention costs were estimated at £59,200. The resulting ICER for the HRLA programme is (59,200 - 619)/0.0233 = £2,514,000 per QALY compared to the control, and (59,200 – 1032)/0.0388 = £1,499,000 compared to no intervention.

### Diet and physical activity

Five studies evaluated an intervention aiming to improve diet and exercise levels in a marginalised community using culturally tailored approaches [78-82]. Keyserling *et al.*[78] described an intervention for women with diabetes, while the four other studies [79-82] were aimed at healthy adults. Each intervention involved community health workers or promotoras delivering individual counselling and advice with the goal of increasing exercise and/or improving diets.

The evidence of improvements in diet, based on self-reported intake, is undermined by evidence of under-reporting of consumption. There is evidence of improvement in fat intake and fruit and vegetable consumption, but studies measuring weight showed no change. Physiological evidence reported in Staten *et al.* is mixed [79]. Keyserling *et al.* reports no improvements in HbA1c levels despite extensive health advice and counselling [78]. The evidence of unreliability of self-reported dietary intake might also lead us to doubt the self-reported evidence of increased physical activity. Elder 2006 reports that improvements from the intervention observed at three months dissipated at six and 12-month follow-up [80].

Overall, the physiological data conflict with the self-reported improvements in diet or exercise, and there is evidence that any possible changes are not sustained. These results suggest that individual counselling from HRLAs is ineffective at achieving long-term changes in diet or exercise patterns. Consequently, these interventions do not appear to be cost-effective.

### Breastfeeding

Two studies concerned breastfeeding: the study by Dennis *et al.*[83] took place in Canada in circumstances similar to a UK context, while the study by Morrow *et al.*[84] was conducted in Mexico. The Canadian intervention utilised telephone support from a woman experienced with breastfeeding, in addition to standard practice. The study reported risk ratios at four, eight and 12 weeks for increased breastfeeding attributable to the programme. The risk ratio at six weeks was estimated at 1.12 (95% confidence interval: 1.01 – 2.00) using regression and combined with UK data on breastfeeding [85] to estimate the peer support programme could increase breastfeeding at six weeks in the UK from 48% to 54%.

Reported benefits of breastfeeding include gains in Intelligence Quotient [86] and reductions in obesity [87] and childhood type 1 diabetes [88]. We did not, however, find any studies estimating a gain in QALYs from breastfeeding. Consequently, we could not estimate an ICER for the HRLA intervention described in Dennis *et al*.

### Mental health promotion

Only one study was identified that addressed mental health issues [89]. The intervention involved an experienced parent providing support to families of children with selected chronic diseases. The control group were given a telephone number through which they could access the HRLA, but only two mothers called the number, suggesting selective demoralisation occurred in the control arm.

The study reported a statistically significant improvement in anxiety levels of 2.1 points on the Psychiatric Symptom Index (range 0 to 100) [90] attributable to the intervention. Costs were not reported but the intervention consumed ten hours of contact time plus telephone support from paid peer advisers, in addition to support from a clinical specialist. Hence costs were likely to be large. We found insufficient evidence in the literature to attach a QALY gain to reductions in anxiety. Hence, we did not calculate an ICER. The change in anxiety appears to be modest and the comparator used in the trial may have biased this estimate. Given the resource intensive nature of the intervention it is unlikely to be cost-effective.

## Discussion

### Main findings of this study

Our analysis suggests that peer- or lay-delivered smoking cessation interventions are highly cost-effective compared to no intervention. Whilst smokers’ clinics are more cost-effective they may not be accessed by marginalised groups. Promotion of uptake of mammography, exercise and healthy eating, or HIV prevention activities by HRLAs are not cost-effective. Programmes directed towards improved chronic disease management have the potential to be cost-effective, particularly for patients with type 2 diabetes. The conclusions on physical activity and healthy eating flow from a lack of evidence of effectiveness in these areas. Where there is evidence of effectiveness, lay health advisers are not always cost-effective. The key driver is the size of the potential health gain from the behaviour promoted. This is large for smoking cessation, and justifies a relatively intensive intervention. The gain from mammography, recently estimated to be an additional 9.2 days life expectancy [91], is simply insufficient to justify even a low intensity promotion programme. The benefits from improved management of diabetes are potentially large, and may justify a low intensity intervention. Whilst the benefit of averting HIV infection is large, the health gain from reducing needle-sharing is modest, and consequently HIV risk reduction programmes are unlikely to be cost-effective in the UK. We were unable to link evidence of effectiveness of HRLA interventions in mental health and breastfeeding promotion with changes in HRQoL.

### Strengths and limitations of this study

This analysis is based on the first systematic review of the diverse range of HRLA roles to be found in developed countries [15]. The review was comprehensive both in its scope and in the focus on acceptability, effectiveness and cost-effectiveness of interventions. Nevertheless, we found few studies assessing outcomes after one year. Restrictions on inclusion by study design and quality assessment excluded many weaker studies. The remaining studies were predominantly single focus interventions, with defined and often standardised protocols, and a predominant focus on individual behaviour change rather than community development. The small number of studies reviewed in each area raises the possibility of publication bias leading to an over-estimate of the effectiveness of HRLA programmes. We lacked sufficient evidence to determine whether any of the interventions reduced or widened health inequalities. However, effective interventions targeted at marginalised communities are likely to reduce the socio-economic gradient in unhealthy behaviours.

The benefits of lifestyle interventions are typically accrued decades into the future for younger people, hence capturing long term outcomes is challenging. Consequently, we had to model long term impact from surrogate short term outcomes which raises concerns about whether the observed behaviour changes were maintained. In the absence of evidence we applied conservative assumptions on the maintenance of behaviour changes rather than simply assuming that no relapse occurred. Nevertheless, our conclusions on mammography promotion were robust to the very generous assumption, commonly applied, that increased adherence is maintained for life. Data on long term maintenance would greatly improve the quality of evaluations of health promotion programmes. We also selected conservative estimates of the health gain from lifestyle changes with regard to our resulting inference – high estimates for mammography and low estimates for smoking cessation – and we explored the impact of changing assumptions with sensitivity analysis. Inferences around mammography promotion and management of type 2 diabetes were robust to this analysis.

### Interpretation of findings and relationship to existing knowledge

Few studies have evaluated the cost-effectiveness of HRLA interventions. Of the three studies we found which did, [31,36,56] two had methodological weaknesses [36,56]. Andersen *et al.* appear to make the commonly applied but unrealistic assumption that behaviour change observed during follow-up is maintained for life [56]. In contrast, assumptions of cost increases from improved management of type 2 diabetes made by Mason *et al.* in the evaluation of the telephone based counselling intervention appear unrealistic [36]. We drew on the approach of Mason *et al.*, but sought to apply the most appropriate estimates of the costs and effects of behaviour changes along with more realistic assumptions on adherence. With the exception of smoking cessation, there was very little evidence to guide assumptions on adherence and so we tested our assumptions in sensitivity analysis. This study is the first to evaluate the cost-effectiveness of HRLA interventions across a diverse range of health behaviours using the same methodology and applying conservative assumptions on adherence. The results indicate that HRLAs can be cost-effective when they target behaviours associated with significant detriments to health.

### Conclusion and implications

Cost-effectiveness of HRLAs are strongly influenced by the size of the health gain from the lifestyle changes targeted. For example, lifetime adherence to mammography screening yields health gains of around 0.04 QALYs compared to 0.4 QALYs for a person with type 2 diabetes reducing their glycosylated haemoglobin by 1%, and 2.0 QALYs for smoking cessation. Consequently the potential health gains from interventions that promote mammography are small compared to those promoting smoking cessation. HRLAs are likely to be most profitably employed delivering interventions with the potential to generate large health gains. They are unlikely to be cost-effective when targeting behaviours with only very modest potential to increase expected HRQoL (such as attendance at mammographic screening). Further research on adherence to short term behaviour changes might facilitate more informed assumptions on the long term maintenance of behaviour changes which underpin estimates of the cost-effectiveness of interventions aimed at improving unhealthy lifestyles.

## Abbreviations

HRLA: Health related lifestyle adviser; QALY: Quality adjusted life year; ICER: Incremental cost-effectiveness ratio; HRQoL: Health related quality of life; HIV: Human immunodeficiency virus.

## Competing interests

The authors declare that they have no competing interests.

## Authors’ contributions

SC and MW conceived the study; CD and MP designed the study economics aspects; SC, ML, NF, KD, LG and SV undertook the systematic review; MP undertook the analysis; MP, SV and SC wrote the manuscript; MW, CD, KD, NF and ML commented on and revised drafts; SC is guarantor. All authors read and approved the final manuscript.

## Authors’ information

Cost-effectiveness and Resource Allocation (IF=1.68) – Instructions for authors:

http://www.resource-allocation.com/authors/instructions/research.

## Supplementary Material

Additional file 1 Estimated costs and calculations of cost-effectivenesss of interventions. Click here for file
