# Supplementary material for: Cost-effectiveness of health-related lifestyle advice delivered by peer or lay advisors: synthesis of evidence from a systematic review
Source: Cost Eff Resour Alloc. 2013 Dec 4;11:30. doi: 10.1186/1478-7547-11-30 (PMC4175508; doi:10.1186/1478-7547-11-30)
Supplement: Additional file 1 — Estimated costs and calculations of cost-effectivenesss of interventions. [file 1478-7547-11-30-S1.docx]

**ADDITIONAL FILES**

**Additional file 1 – estimated costs and calculations of cost-effectivenesss of interventions**

***Management of type 2 diabetes***

The intervention in Gary 2003[33] appears to have employed a full time nurse and a community health worker (CHW) for the two year duration of the intervention, with the CHW spending half of his/her time with the participants in the CHW arm. A yearly cost of £31,043 was assumed for the CHW based on the cost of a social work assistant,[20] giving a per participant cost of £757. Lujan 2007[34] reports that two promotoras provided the 3 month intervention to groups. It seems unlikely that these ran concurrently, hence it was assumed that the promotoras were employed for a year. In addition transport was provided for the participants. Applying the same cost estimated for the CHW to the promotoras gives estimated staff costs of £62,086 for the year. It was assumed that 50% of the participants utilised the transport with costs of £20 per trip, giving total transport costs of £12,000. Hence the overall cost was estimated at £74,086 (£988 per participant).

Calculation of ICER for Young 2005[32] and Lujan 2007[34]

| Intervention | Per participant cost | Discounted 10 year cost* | Mean fall in % HbA1c | Costs averted | Inc. cost | QALYs gained | Inc. QALY gain | ICER |
| --- | --- | --- | --- | --- | --- | --- | --- | --- |
| Young | 258 | 905 | 0.31 | 106 | 799 | 0.0589 | 0.0589 | 13,565 |
| Lujan | 988 | 3467 | 0.45 | 154 | 2514 | 0.0855 | 0.0266 | 94,511 |

All costs are in 2008 £s. Inc. = Incremental. *Discounted ten year costs is the sum of costs in year 1 (undiscounted), year 3, year 6 and year 10.

***Smoking cessation***

The intervention in Woodruff 2002[45] consisted of four home visits of 1–2 hours’ duration and 3 telephone calls (15–30 minutes), giving a total time of around 7 hours. Mean participation was 3.5 sessions. We applied a rate of £31 per hour (alcohol health worker[20]) to an estimate of 3.5 hours’ contact time to give a cost of £109 per participant. We also assumed that recruiters were paid £20 per participant and that the intervention required co-ordination by a full-time employee for three months (estimated at £13,413 based on the cost of a social work team leader including overheads[20]). The total costs were therefore estimated at £33,537 for 156 participants, or £215 per participant, which is in line with costs per participant of US$300 reported in Emmons *et al.*[44]

***Mammography promotion***

Calculation of ICER for Andersen 2002[56] and Paskett 2006[58]

| Intervention | Programme cost per participant (£) | Discounted 10 year programme cost (£)* | Mean % increase in users | Additional costs from screening (£) | Inc. Cost (£) | QALYs gained | Inc. QALY gain | ICER |
| --- | --- | --- | --- | --- | --- | --- | --- | --- |
| Andersen | 34 | 119 | 1.25 | 1.85 | 121 | 0.0005 | 0.0005 | 251,000 |
| Paskett | 657 | 2306 | 7.60 | 11.25 | 2196 | 0.0025 | 0.0020 | 896,000 |

All costs are in 2008 £s. Inc. = Incremental. *Discounted ten year costs is the sum of costs in year 1 (undiscounted), year 3, year 6 and year 10.

***HIV prevention***

Dickson-Gomez 2003[66] reports compensation for participants of $20 for completing the baseline interview and $15 for each of the ten two hour training sessions they attended. Two thirds of the 250 participants were randomised to the intervention (approx. 168). The training sessions were conducted in small groups. Assuming the mean group size was 7 this would require training 24 groups. With the associated administration this is likely to require a full time employee for one year. The total yearly cost of an alcohol health worker (£47,317) was applied.[20] Assuming mean attendance is 5 sessions the participant remuneration costs are $(20 + (15 x 5)) x 168 = $15,960 or £11,800 (‘2008’ GBP). Hence implementing the programme would costs a minimum of £59,200.

A small proportion of participants (22, 19% of injectors) reported sharing needles at baseline. We estimated 12 incidences of needle-sharing per participant who shared needles over six months. At review, 69% of HRLA intervention recipients reported reductions in unhygienic needle practices compared to 30% in the control group. The number of needle sharers in the intervention arm is not reported but participants were randomised in the ratio 2:1 between the intervention and control arm, giving an estimate of 10 participants reducing unsafe practices in the intervention. The corresponding estimate of participants reducing unsafe practices after applying the effectiveness of the control programme is 4. The extent to which unhygienic needle practices were reduced is not quantified. Applying a conservative assumption of a 25% reduction in incidences of needle-sharing for those who reported behaviour change gives a change from 12 to 9 incidences of needle sharing over the six-month intervention period.

From equation A in the main text the probability of infection is reduced by 0.002891-0.002169 = 0.000722 for a user reducing risky behaviour (from n=12 to n=9). For the 10 users who report behaviour change after the intervention, the number of HIV cases averted is 0.000722 x 10 = 0.00722 cases. Application of the control programme would have averted 0.000722 x 4 = 0.002888 cases. The additional gain from the programme is therefore an additional 0.004332 cases averted.

Dominated strategies yield lower health gains at higher cost than alternatives. Extendedly dominated strategies yield lower health gains at higher cost than some combination of two alternative strategies.
